# Ultrasound-Assisted Preparation, Characterization, and Antibacterial Activity of Montmorillonite Modified by ε-Polylysine Hydrochloride

**DOI:** 10.3390/ma12244148

**Published:** 2019-12-11

**Authors:** Xinfu Yuan, Jinli Zhang, Rui Zhang, Jingyuan Liu, Wentao Wang, Hanxue Hou

**Affiliations:** 1 College of Food Science and Engineering, Shandong Agricultural University, Engineering and Technology Center for Grain Processing of Shandong Province, Tai’an 271000, China; KL2yxf1234@163.com (X.Y.); zhangli9538@sina.com (J.Z.); xuyuyisha@163.com (R.Z.); LJY1664961793@126.com (J.L.); 2 State Key Laboratory of Biobased Material and Green Papermaking, Qilu University of Technology, Shandong Academy of Sciences, Jinan 250000, China

**Keywords:** ε-polylysine hydrochloride, montmorillonite, ultrasonic treatment, antibacterial activity

## Abstract

In this study, two types of antibacterial montmorillonites (Mt) were prepared using a facile method. The Mt modified with ε-polylysine hydrochloride (ε-PL) was named PL-Mt, while the Mt dually modified with dioctadecyl dimethylammonium chloride (D1821) and ε-PL was named PL-OMt. The results of the X-ray diffraction, Fourier-transform infrared (FTIR) spectroscopy and thermogravimetric analysis (TGA) of the PL-Mt indicated that 30% ε-PL was the most suitable amount for intercalating the Mt. The particle size and distribution of the ε-PL in the solution demonstrated that the Mt d-value could not be further increased owing to the increasing ε-PL diameter. The result of the X-ray diffraction of PL-OMt displayed that ultrasonic treatment at 600 W facilitated ε-PL to intercalate into the OMt interlayer space. The PL-OMt prepared with ultrasonic treatment at 600 W exhibited antibacterial activity against *Escherichia coli* and *Bacillus subtilis* superior to that of the PL-OMt prepared with higher-power ultrasonic treatment. Thus, the addition of 30% ε-PL based on the dry Mt mass is the most suitable ratio for preparing PL-Mt, while ultrasonic treatment at 600 W is the most suitable for preparing PL-OMt. These findings may expand the application fields of ε-PL.

## 1. Introduction

Montmorillonite (Mt), which is a type of 2:1 layered silicate, is widely used owing to its environmentally friendly nature, natural abundance, low cost and unique structure and properties [1,2,3,4]. In recent years, Mt has attracted increasing attention, not only in enhancing mechanical, barrier and optical properties [5,6], but also in acting as a carrier of antibacterial agents in biopolymers for food packaging [7]. As Mt does not exhibit antibacterial activity in nature, it requires modification to play the role of an antibacterial agent in biodegradable materials. Lavorgna [8] prepared multifunctional bionanocomposites by loading the chitosan matrix with silver-Mt antimicrobial nanoparticles obtained by replacing the Na^+^ ions of raw Mt with Ag^+^ ions. Makwana [9] investigated the effects of silver-modified Mt on the physical, mechanical and antimicrobial properties of carboxymethylcellulose bionanocomposite films. Nouri [7] prepared Mt−copper oxide nanocomposites to enhance the optical, mechanical and antibacterial properties of chitosan films. Although metals or metal oxides have strong antibacterial activity, the possible side effects of silver nanoparticles on human health are mostly unknown, and constitute a topic of dispute and concern [10], and similar safety issues exist for Cu^2+^ ions [7].

Thus, substitution of metals with an antibacterial agent which offers both a high level of safety and relatively strong antibacterial activity may be a better choice.

In Japan, ε-polylysine hydrochloride (ε-PL) has entered the commercial market as a natural antimicrobial food additive. Numerous foods contain ε-PL with different contents, such as fish sushi (1 to 5 mg/g), boiled rice and cooked vegetables (0.01 to 0.5 mg/g) and other foods [11,12]. The safety of ε-PL as a food additive was authorized by the Food and Drug Administration and the National Health Commission of the People’s Republic of China in 2003 and 2014, respectively [12,13,14]. Moreover, ε-PL can be decomposed into lysine in the human body and can serve as a lysine source. Therefore, it is a nutritive antibacterial agent.

ε-PL molecules are cationic surfactants owing to their positively-charged amino groups in water. According to growth inhibition studies using yeast, fungi and Gram-positive or Gram-negative bacterial species, ε-PL has a wide antimicrobial spectrum [15]. It can destroy the cell wall structure through the interaction between the positively- and negatively-charged residues on the surface of bacterial cells, thereby leading to cell death and inhibiting microbial contaminants [16,17]. ε-PL is a cationic homopolyamide of 25–30 lysine residues with an amide linkage between the ε-amino and α-carboxyl groups [18,19]; therefore, it has the capacity of ion exchange with the Na^+^ ions of Mt. Compared to other antibacterial agents, the above-mentioned advantages make ε-PL a strong candidate for designing a new type of antibacterial Mt, which is superior to the commonly used antibacterial Mt modified with metals or metal oxides.

Mt is previously functionalized with surfactants or silane coupling agents to make them more miscible with organic and polymeric materials, resulting in better dispersion and thermal properties [10,20]. Lipophilicity of organic modified Montmorillonite (OMt) makes them suitable materials for incorporating drug molecules and slowing their release [21]. Dioctadecyl dimethylammonium chloride (D1821) is an organic surfactant with double-alkyl-chain and higher molecular weight. These features of D1821 will facilitate more D1821 to enter the Mt interlayer space than the other single-alkyl-chain quaternary ammonium salts, resulting in a lager d-value of OMt [22], and then the larger d-value might facilitate ε-PL to intercalate into the OMt interlayer space. In addition, researchers reported that ultrasonic treatment can be used to locally destroy the long-range order of Mt interlayer space and allow more modifier to intercalate into it. Chaudhary [23] found that the d-value of the Mt expanded to accommodate the various modifiers after sonicating. Thus, as a nanofiller and antibacterial agent, when PL-OMt is introduced into the biodegradable polymer matrix by the extrusion process, the mechanical and barrier properties, as well as antibacterial activity, will be improved. The preparation of antibacterial Mt and its properties have been reported. However, to the best of the authors’ knowledge, little information is available on the preparation and antimicrobial activity of Mt modified with ε-PL. Therefore, the aim of this study was to (1) prepare two types of antibacterial Mt, where one is PL-Mt (Mt modified with ε-PL), and another is PL-OMt (Mt dually modified with D1821 and ε-PL, assisted by ultrasound treatment), and (2) to investigate the structures and physicochemical properties of PL-Mt and PL-OMt, and (3) to compare the antibacterial activities of these two types of antibacterial Mt.

## 2. Materials and Methods

### 2.1. Materials

Sodium Mt (Na^+^-Mt) was purchased from Zhejiang Fenghong New Material Co., Ltd. (Huzhou, Zhejiang, China). D1821 (purity > 99%) was provided by Shandong Xiya Chemical Industry Co., Ltd. (Jinan, Zhejiang, China). The ε-PL was obtained from Zhejiang Silver-Elephant Bio-engineering Co., Ltd. (Taizhou, Zhejiang, China). The phosphate buffer solution powder was provided by Beijing Zhongshan Golden Bridge Biotechnology Co., Ltd. (Beijing, China). The yeast extract and tryptone were purchased from Beijing Aoboxing Biotechnology Co., Ltd. (Beijing, China). Nutrient agar was purchased from Qingdao Hope Bio-Technology Co., Ltd. (Qingdao, Shandong, China).

The test strains, Gram-negative bacteria *Escherichia coli* (*E. coli*), and Gram-positive bacteria *Bacillus subtilis* (*B. subtilis*) were provided by the Agricultural Culture Collection of China. All other chemical reagents were analytical grade and were purchased from Kaitong Chemical Reagent Co., Ltd. (Tianjin, China).

### 2.2. Preparation of PL-Mt

Na^+^-Mt (20 g) was dispersed in 300 mL of deionized water and magnetically stirred for 12 h at 25 °C to obtain a Mt dispersion (the stirring rate was consistent and kept at 600 rpm all the time). The ε-PL was dissolved in 100 mL of deionized water at 25 °C. The ε-PL/Mt mass ratios were 0/100, 10/100, 30/100, 50/100 and 70/100, based on the dry weight. The ε-PL solution was poured into the Mt dispersion and mechanically stirred at 60 °C for 3 h. The Mt modified with ε-PL was denoted by PL-Mt, while the PL-Mt modified with 10%, 30%, 50% and 70% ε-PL was denoted by PL-Mt10, PL-Mt30, PL-Mt50 and PL-Mt70, respectively. The PL-Mt was collected by centrifuging and washing with deionized water several times until the supernatant was free from chloride anions, as determined by an AgNO_3_ test. Finally, the PL-Mt was dried in an oven at 60 °C overnight and sieved through a 200-mesh sieve.

### 2.3. Preparation of PL-OMt

A total of 20 g of Na^+^-Mt was dispersed in 300 mL of deionized water and magnetically stirred for 12 h at 25 °C (the stirring rate was consistent and kept at 600 rpm all the time), followed by continuous sonication at various power values (0, 600, 1200 and 1800 W) for 3 min. According to a previous study [24], 6 g of D1821 was dissolved in 50 mL of deionized water at 80 °C. The D1821 solution was poured into the Mt dispersion and mechanically stirred at 80 °C for 3 h to obtain the OMt dispersion. Furthermore, 6 g of ε-PL (30/100 based on the Mt dry weight) was dissolved in 100 mL of deionized water at 25 °C, poured into the OMt dispersion, and mechanically stirred at 60 °C for a further 3 h. The Mt modified with D1821 and ε-PL was denoted by PL-OMt; the corresponding PL-OMt was referred to as 0W-PL-OMt (without ultrasonic treatment), 600W-PL-OMt, 1200W-PL-OMt and 1800W-PL-OMt, respectively. The PL-OMt was collected by centrifuging and washing several times with deionized water until the supernatant was free from chloride anions, as determined by an AgNO_3_ test. Finally, the PL-OMt was dried in an oven at 60 °C overnight, and ground through a 200-mesh sieve.

### 2.4. X-ray Diffraction (XRD) Analysis

The X-ray Diffraction (XRD) analysis of the Na^+^-Mt, PL-Mt, OMt and PL-OMt were performed on a D8 Advance X-ray diffractometer (Bruker AXS, Karlsruhe, Germany) with CuKα radiation (λ = 1.5406 Å) over a diffraction angle range of 2θ = 1°–10° at 0.02°/s. The basal reflection (d-value) of the samples was calculated by Bragg’s equation, λ = 2dsinθ, where θ is the diffraction angle and λ is the X-ray radiation wavelength.

### 2.5. Particle Size and Distribution of ε-PL

The mean diameter and distribution of the ε-PL solutions of different concentrations were determined by the NanoBrook ZetaPlus Potential Analyzer (Brookhaven Instruments Corporation, New York, NY, USA). All measurements were performed in triplicate (n = 3), and the standard deviation (SD) was recorded.

### 2.6. Fourier Transform Infrared (FTIR) Spectroscopy

The Fourier Transform Infrared (FTIR) spectra of the Na^+^-Mt, PL-Mt, OMt and PL-OMt were analyzed by a Nicolet iS5 spectrometer with iD5 ATR sampling accessory over the wavelength range 600–4000 cm^−1^ (Thermo Fisher Scientific, Waltham, MA, USA). The resolution was 4 cm^−1^ and the number of accumulated scans was 32. The sample was mounted directly into the sample holder.

### 2.7. Thermogravimetric Analysis (TGA) 

The thermogravimetric analysis (TGA) of the ε-PL, D1821, Na^+^-Mt, PL-Mt, OMt and PL-OMt was performed on a Shimadzu TA-60 (Kyoto, Japan). Approximately 5 to 10 mg of the sample was heated in a platinum crucible from 30 to 600 or 800 °C at a heating rate of 10 °C/min in a high-purity nitrogen flow of 50 mL/min.

### 2.8. Determination of Antibacterial Activity of Modified Mt

The antibacterial activity of the Mt, PL-Mt30, 1800W-OMt and PL-OMt powders was determined using the E2149 test of the American Society for the Testing and Materials (ASTM), according to previous studies with some modifications [25,26,27,28,29]. The Gram-negative bacteria *E. coli* and Gram-positive bacteria *B. subtilis* were used as the reference strains and a bacterial dispersion with a density of 10^8^ CFU/mL was prepared in the experiments. An amount of 1 mL of the bacterial dispersion was pipetted into a test tube containing 9 mL of sterilized water and mixed thoroughly, and the process was repeated to prepare 10-fold serial dilutions. Finally, a bacterial dispersion with a density of 10^3^ CFU/mL was obtained. The phosphate buffer solution powder (11.74 g) was dissolved in 1000 mL of sterilized water at approximately 30 °C (0.01 mol/L, pH = 7.3). The Mt, PL-Mt30, 1800W-OMt and PL-OMt powders of 1.0 g were added to separate flasks, each containing a test culture of 1 mL and buffer solution of 19 mL. An inoculum of 1 mL of bacterial dispersion and buffer solution of 19 mL in a flask with no test substance was used as a control. Thereafter, the flasks were shaken for 8 h at 150 rpm (37 °C) in an orbital shaker. A 100-μL aliquot was obtained from these solutions, transferred to a Petri dish containing approximately 20 mL of nutrient agar, and incubated for 24 h at 37 °C to count the colonies. All experiments were repeated at least twice to verify the reproducibility.

### 2.9. Statistical Analysis

Microsoft Excel 2016 and the SPSS software (version 20, IBM Corporation, New York, NY, USA) were used for the statistical analyses. The data were subjected to analysis of variance (ANOVA). Comparisons of the mean values of the mean diameters and polydispersity index (PDI) were carried out by Duncan’s multiple-range test with *p* < 0.05.

## 3. Results and Discussion

### 3.1. Characterization of PL-Mt

#### 3.1.1. XRD Analysis of PL-Mt

**Figure 1:**
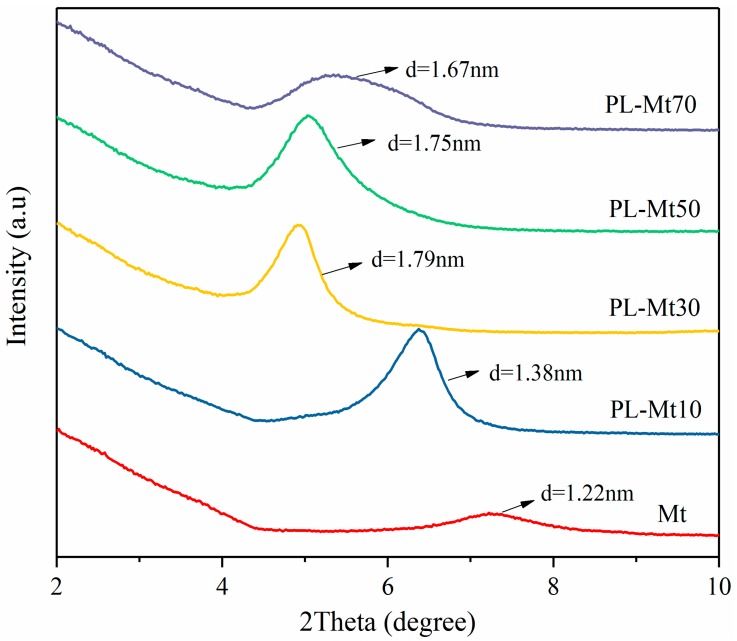
X-ray Diffraction (XRD) patterns for Mt modified with ε-PL at various amounts.

XRD is an important tool for analyzing the structures of nanocomposites with Mt. This technique provides information regarding the position, shape and intensity of the basal structure of the layered silicate [30,31]. The XRD patterns of the PL-Mt and Mt are displayed in Figure 1. The 001 reflection of the Mt was located at 7.26°, and the d-value of the 001 reflection of the Mt was 1.22 nm according to Bragg’s equation (Figure 1). The XRD curves of the PL-Mt with different amounts of ε-PL exhibited similar trends to those of a previous report [24]. All of the reflection peaks of the PL-Mt shifted to lower 2θ angles, which proved that the ε-PL successfully intercalated the interlayer space of the Mt and contributed to the increase in the d_001_-value. The d_001_-values of the PL-Mt10, PL-Mt30, PL-Mt50 and PL-Mt70 increased to 1.38, 1.79, 1.75 and 1.67 nm, respectively, from the original 1.22 nm of the Mt. The d-value of the PL-Mt decreased with increasing amounts of ε-PL when the addition amounts exceeded 50%. This may be owing to the increase in the ε-PL diameters at high concentration, which inhibited ε-PL from entering the Mt interlayer space, and this apparently did not allow for a further increase in the d-value with an increasing modifier amount [23].

#### 3.1.2. Determination of Diameters and Distribution of ε-PL in Solutions

The mean diameters of the ε-PL in the solutions of different concentrations varied substantially. The mean particle size of the ε-PL gradually increased with an increasing concentration. Specifically, the average diameters of the ε-PL in the solutions with concentrations of 1%, 3%, 5% and 7% were 1134.7, 1218.5, 1413.8 and 1579.3 nm, respectively (Table 1). The PDI is an indicator of the distribution and homogeneity of dispersed particles. A smaller PDI results in a narrower particle size distribution [32]. The PDIs of the 1%, 3%, 5% and 7% ε-PL solutions were 0.211, 0.181, 0.152 and 0.146, respectively, suggesting that the ε-PL solutions of 5% and 7% exhibited superior uniformity and a narrower particle size distribution than those of the former two. That is, the particle sizes of the 5% and 7% ε-PL solution were closer to their mean diameters. These two results can integrally account for the fact that the d_001_-values of the PL-Mt50 and PL-Mt70 were lower than that of the PL-Mt30, which is in accordance with the above speculation from the XRD studies.

#### 3.1.3. FTIR Analysis of PL-Mt

**Figure 2:**
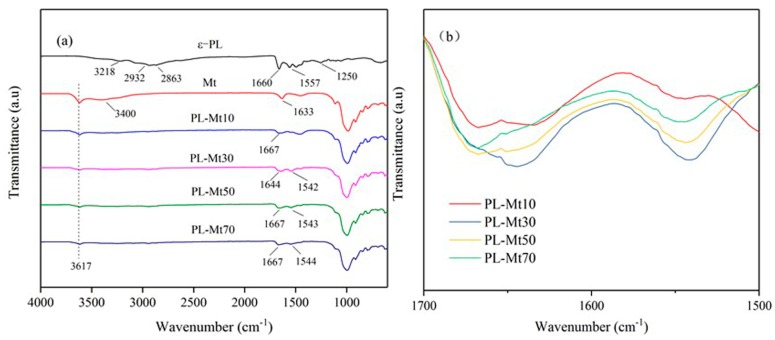
Fourier-transform infrared (FTIR) spectra of ε-PL, Mt, PL-Mt10, PL-Mt30, PL-Mt50 and PL-Mt70 (**a**); magnification of 1700 to 1500 cm^−1^ range of PL-Mt (**b**).

FTIR spectra can be used to identify functional groups and can illustrate the different vibrational modes of various bonds [33]. As illustrated in Figure 2a, the typical FTIR spectra of the ε-PL absorbance was observed at 3218, 2932, 2863, 1660, 1557 and 1250 cm^−1^, which could be attributed to the NH_2_ symmetric stretching vibration, antisymmetric and symmetric stretching vibration of methylene (–CH_2_–), C=O stretching vibration (amide I), N–H bending (amide II) and C–N stretching vibration, respectively [14,34]. The characteristic peak of the Mt was exhibited at 3617 cm^−1^, which is known as the O–H stretching vibration of Al–OH and Si–OH [35]. The peaks at 3400 and 1633 cm^−1^ could be ascribed to the O–H stretching and bending vibrations, respectively [36]. Compared with the spectrum of ε-PL, characteristic peaks were detected at 1644 cm^−1^ and 1542 cm^−1^ in the spectrum of PL-Mt30, and detected at 1667 cm^−1^ and 1543 cm^−1^ in others PL-Mt. The results verified that the ε-PL was successfully interacted with the Mt and prepared a new type of antibacterial PL-Mt [34]. Furthermore, with increasing amounts of ε-PL, the intensity of C=O stretching vibration (amide I) of the PL-Mt increased and reached the maximum for PL-Mt30, as indicated in Figure 2b. Therefore, it can be concluded that the amount of 30% ε-PL is the most suitable ratio for intercalating Mt. 

#### 3.1.4. TGA of PL-Mt

**Figure 3:**
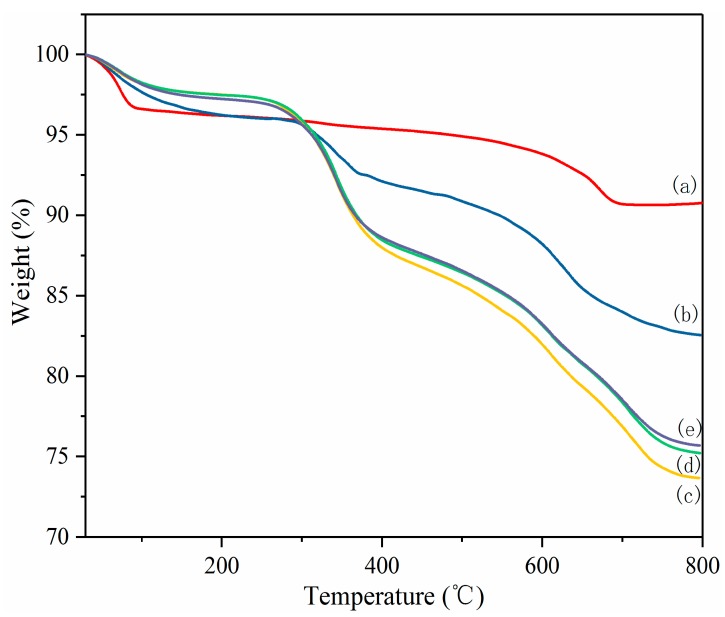
Thermogravimetric analysis (TGA) of Mt (**a**), PL-Mt10 (**b**), PL-Mt30 (**c**), PL-Mt50 (**d**) and PL-Mt70 (**e**).

TGA was used to estimate the intercalation extent of the Mt by ε-PL, as well as the thermal stability of the Mt, ε-PL and PL-Mt [37]. As illustrated in Figure 3, the Mt exhibited excellent thermal stability, with a residue as high as 90.78% at 800 °C, and a pronounced mass loss from ambient to 100 °C owing to the evaporation of absorbed water. The mass loss values were mostly constant in the temperature range of 100–600 °C. Above 600 °C, the mass decreased again as a consequence of dehydroxylation of the Mt [31,36].

The TGA curve of the ε-PL is displayed in Figure 3. In this case, at 30–130 °C, the first stage of the mass loss could also be ascribed to the desorption of the absorbed water. Moreover, the first-stage mass loss of the ε-PL was substantially higher than that of the Mt, because ε-PL easily absorbs moisture [21]. The second mass loss stage of the ε-PL started at approximately 300 °C and a residue of only 7.43% was exhibited at approximately 600 °C. The results demonstrated that the Mt had a higher thermal stability than the ε-PL, and the mass loss of the PL-Mt is between the Mt and ε-PL. 

As also indicated in Figure 3, all the PL-Mt samples exhibited four mass loss steps. The first mass loss step, in the temperature range 30–150 °C, was mainly attributed to the desorption of absorbed water on the Mt. The second mass loss step, at approximately 220–410 °C, corresponded to the thermal decomposition of the ε-PL intercalating the Mt [38]. The third and fourth steps, in the temperature ranges from 420 to 640 °C and from 650 to 800 °C, respectively, were owing to the decomposition of the remaining ε-PL in the second step and the dehydroxylation of the Mt hydroxyl groups [36].

The total mass losses of the PL-Mt10, PL-Mt30, PL-Mt50 and PL-Mt70 were approximately 17.45%, 26.36%, 24.78% and 24.21%, respectively, in the temperature range of 30–800 °C. This result indicates that the most suitable mass ratio (based on the dry mass) of PL/Mt for intercalating Mt is 30/100.

#### 3.1.5. Antibacterial Activity of PL-Mt30

**Figure 4:**
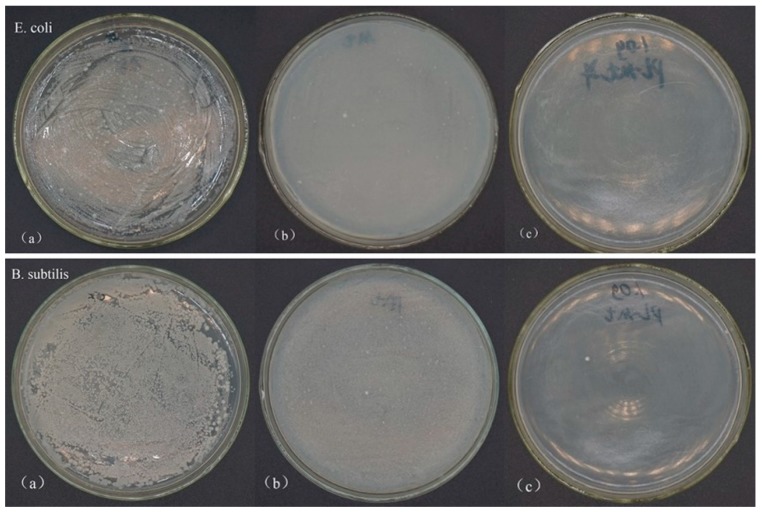
Antibacterial activity of control (**a**), Mt (**b**) and PL-Mt30 (**c**) on *E. coli* and *B. subtilis.*

The antibacterial activities of the raw Mt and PL-Mt30 against *E. coli* and *B. subtilis* were tested in powder form. As can be seen, in the plates of the control group (Figure 4a), the bacteria reached the maximum load on their plates after incubating for 24 h at 37 °C, and the number of surviving bacterial colonies was countless. The proposed mechanism of the inhibitory effect of ε-PL on microbial growth is its electrostatic adsorption to the cell surface of microorganisms, based on its cationic property, followed by the stripping of the outer membrane and abnormal distribution of cytoplasm, and eventually resulting in bacteria death [16,39]. In addition, the result of the FTIR spectrum of PL-Mt30 verified that the ε-PL was successfully interacted with the Mt and prepared a new type of antibacterial PL-Mt. Therefore, the nutrient agar plates containing 1.0 g of PL-Mt30 powder (Figure 4c) displayed that no bacteria survived on them, demonstrating a good antibacterial activity of PL-Mt30 against *E. coli* and *B. subtilis*. This result was in good agreement with a previous report [26] which found that 1.0 g of Ag + OMt powder exhibited good antibacterial activity against *E. coli*. Surprisingly, a few differences can be observed between Figure 4a and Figure 4b. This is because the proportion of the Mt powder might influence the growth of the *E. coli* and *B. subtilis* by means of changing the pH and oxygen concentration of the inoculum, or other factors.

### 3.2. Characterization of PL-OMt

As we all know, the hydrophobic clay materials are compatible with organic materials, such as polymers, drug molecules, oils and hydrocarbons [22,36], and thus it is essential to prepare a type of hydrophobic antibacterial montmorillonite.

#### 3.2.1. XRD Analysis of PL-OMt

**Figure 5:**
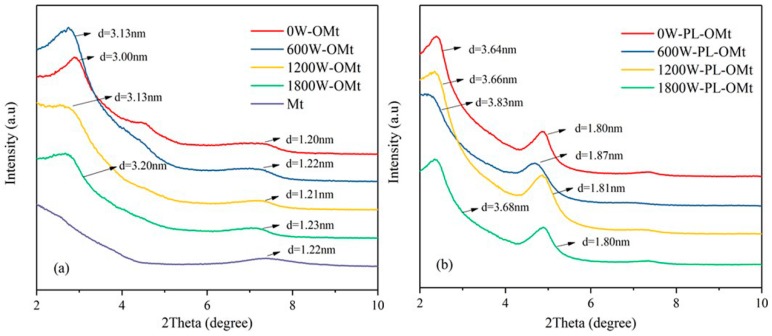
XRD patterns of Mt and OMt (**a**) and PL-OMt (**b**).

The XRD patterns of the Mt and OMt are presented in Figure 5a. The d-value increased with the increase in the ultrasonic power; the highest d-value was 3.20 nm for the OMt prepared at 1800 W ultrasonic power, while the lowest d-value was 3.00 nm prepared at 0 W (without ultrasonic treatment), indicating different extents of the intercalation of the D1821 into the Mt interlayer space [22,40]. Moreover, higher-ordered reflections corresponding to the (002) and (003) planes of the Mt were observed for all of the OMt, suggesting well-ordered layer structures [24].

Figure 5b illustrates the XRD patterns of the PL-OMt based on the OMt and modified by ε-PL at different ultrasonic powers. All of the 001 reflections of the PL-OMt were shifted to lower angles relative to the 001 reflections of the OMt, demonstrating that the ε-PL intercalated the OMt interlayer space [22]. As indicated in Table 2, the specific d_001_-values of the 0W-PL-OMt, 600W-PL-OMt, 1200W-PL-OMt and 1800W-PL-OMt increased to 3.64, 3.83, 3.66 and 3.68 nm, respectively.

According to Figure 5a, the d_001_-values of the 0W-OMt, 600W-OMt, 1200W-OMt and 1800W-OMt were 3.00, 3.13, 3.13 and 3.20 nm, respectively. When subtracting the d_001_-value of the OMt, the ***Δ***d of the 0W-PL-OMt, 600W-PL-OMt, 1200W-PL-OMt and 1800W-PL-OMt were 0.64, 0.70, 0.53 and 0.48 nm, respectively. The ***Δ***d might be representative of the amount of ε-PL intercalated into the layer of OMt after the secondary modification with ε-PL. Compared to the ***Δ***d of the 0W-PL-OMt, this demonstrates that the higher d_001_-value of the 600W-PL-OMt contributed to the intercalation of ε-PL into the OMt layer. When the ultrasonic power increased from 600 to 1200 or 1800 W, the ***Δ***d of the PL-OMt decreased gradually, indicating that the amount of ε-PL entering the OMt interlayer decreased. Therefore, higher ultrasonic power resulted in a lower amount of ε-PL intercalating the OMt interlayer. Furthermore, Figure 5b also illustrates the characteristic peaks at approximately 2θ = 5°, corresponding to the 001 peaks of the PL-Mt30 in Figure 1. This phenomenon proves that the ε-PL successfully intercalated the OMt interlayer space.

#### 3.2.2. FTIR Analysis of OMt and PL-OMt

**Figure 6:**
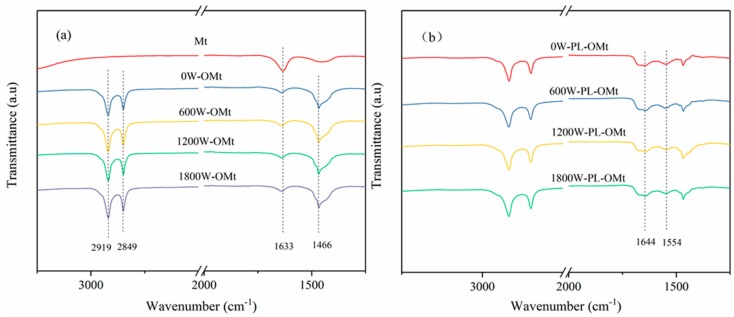
FTIR spectra Mt and OMt (**a**) and PL-OMt (**b**).

The FTIR spectra of the Mt, OMt and PL-OMt are presented in Figure 6. Compared to the raw Mt, three obvious characteristic peaks could be observed at 2919, 2849 and 1466 cm^−1^ in the FTIR spectra of all OMt samples (Figure 6a), which could be attributed to the antisymmetric and symmetric stretching vibrations of the methylene groups (–CH_2_–) of the aliphatic chains of D1821 and the –CH_2_– bending vibrations, respectively [36,41]. Meanwhile, the peak at 1633 cm^−1^ in the FTIR of the raw Mt is ascribed to the –OH bending mode, which became weaker after hydrophobic modification. Compared to the FTIR spectra of the OMt, as indicated in Figure 6b, another two characteristic peaks could be observed at 1644 and 1554 cm^−1^, which are attributed to the C=O stretching vibration (amide I) and N–H bending (amide II) of the ε-PL, respectively [14,34]. The wavenumbers of these two peaks were slightly different for the ε-PL and PL-OMt, indicating that the surfactant molecules were intercalated into the confined interlayer spaces [40].

#### 3.2.3. TGA of Two Modifiers, OMt, and PL-OMt

**Figure 7:**
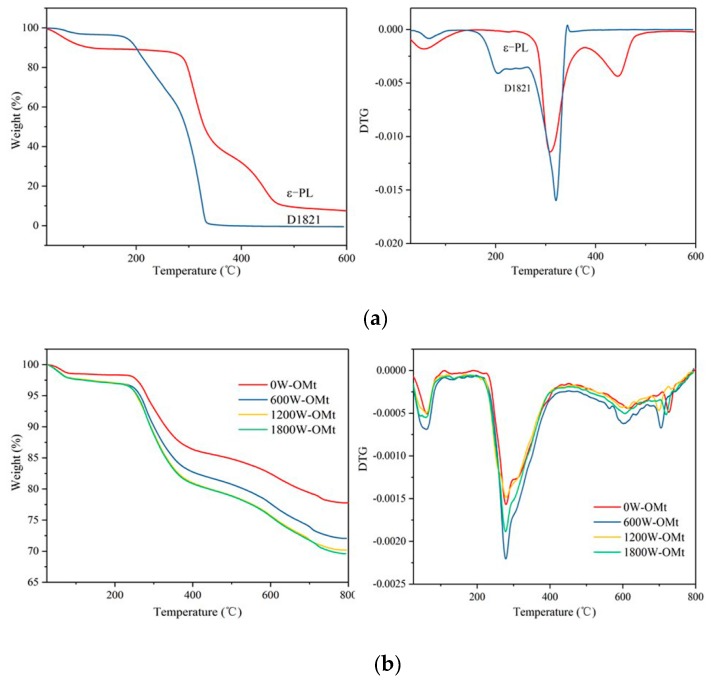

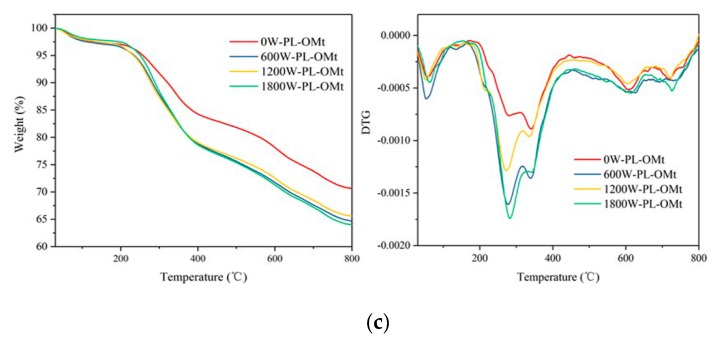
Thermogravimetric analysis (TGA) and differential thermogravimetry (DTG) of ε-PL andD1821(**a**), OMt (**b**) and PL-OMt (**c**).

As depicted in Figure 7, the effects of the simultaneous presence of the D1821 and ε-PL on the thermal properties of the obtained OMt and PL-OMt were evaluated by TGA. Figure 7a indicates that the degradation processes of the two modifiers (ε-PL and D1821) both consisted of three steps. The first step, viz, the degradation within the temperature range from 30 to 150 °C, was attributed to the desorption of adsorbed water of the ε-PL and D1821. The mass losses of the second and third steps were ascribed to the degradation of the ε-PL and D182 themselves. In the second-step mass loss, the D1821 began to degrade at approximately 200 °C and degraded completely above 350 °C, while the ε-PL began to degrade at approximately 300 °C and exhibited a residue of 7.43% at 600 °C, indicating that ε-PL has superior thermal stability to D1821 [42].

Figure 7b illustrates that all the OMt samples exhibited four mass loss stages. The first stage (35–100 °C) was attributed to the desorption of the absorbed water and evaporation of the interlayer water in the Mt. The second stage, at approximately 210–400 °C, was attributed to the thermal decomposition of the loaded D1821 in the Mt [36]. Moreover, the mass losses of the OMt increased with an increase of the ultrasonic power, indicating that higher ultrasonic power contributed to intercalating more D1821 into the Mt interlayer. This result is in line with the above XRD profile (Figure 5a). The third and fourth stages, at temperature ranges 500–650 °C and 650–750 °C, respectively, corresponded to the dehydroxylation of the Mt hydroxyl groups [7,36].

After secondary modification with ε-PL, as indicated in Figure 7c, the most significant difference between the OMt and PL-OMt TGA curves was that the mass loss of the PL-OMt prepared at different ultrasonic power was independent of ultrasonic treatment except for 0W-PL-OMt. That is, the degradation amounts of the PL-OMt prepared at ultrasonic treatment did not change with the increase in the ultrasonic power. This can be explained that the Mt interlayer space was limited, and the total amounts of modifiers that could be accommodated were fixed. Therefore, the amount of ε-PL intercalated into 600W-PL-OMt was superior to that of the PL-OMt prepared with higher-power ultrasonic treatment.

#### 3.2.4. Antibacterial Activity of PL-OMt

**Figure 8:**
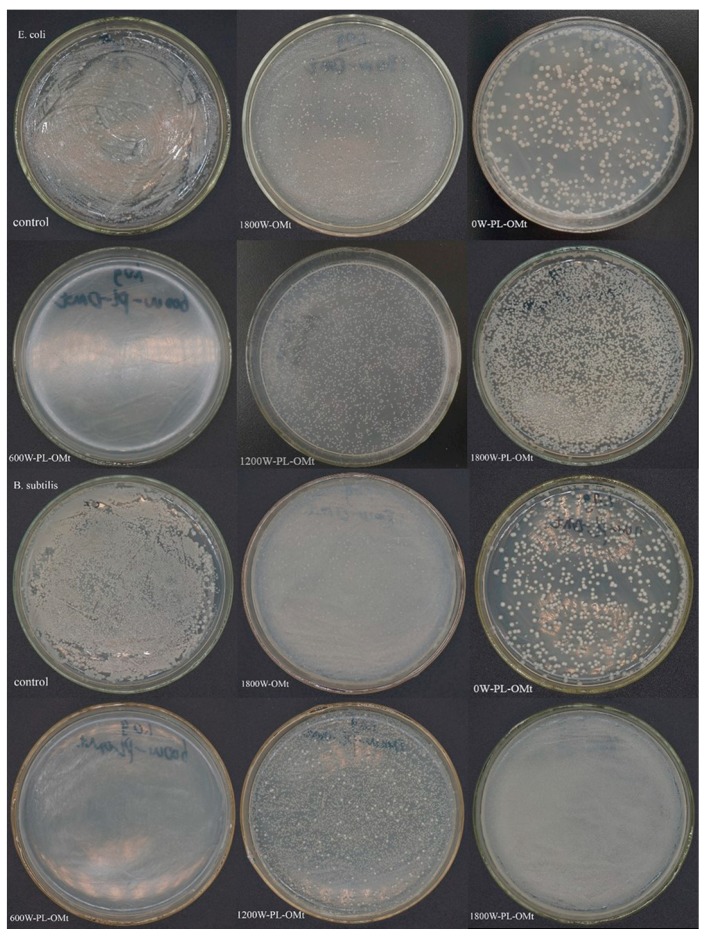
Antibacterial activity of control, OMt and PL-OMt on *E. coli* and *B. subtilis.*

The antibacterial activities of the 1800W-OMt and PL-OMt against *E. coli* and *B. subtilis* were tested in powder form. When the ultrasonic power was 1800 W, the amounts of D1821 intercalated the Mt interlayer space reached the maximum (Figure 5a and Figure 7b), and thus, it was reasonable to select 1800W-OMt as the control for the PL-OMt. As displayed in Figure 8, different antibacterial activities against *E. coli* and *B. subtilis* were obtained in the presence of the PL-OMt and 1800W-OMt. As can be seen from control group plates and 1800W-OMt plates which incubated *E. coli*, the bacteria reached the maximum load after incubating for 24 h at 37 °C, but a few differences can also be observed between them. The PL-OMt prepared at various ultrasonic powers exhibited different antibacterial activity against *E. coli*, and higher antibacterial activity of PL-OMt results in fewer amounts of *E. coli* that survived on its nutrient agar plate. Therefore, lower antibacterial activity than the PL-Mt30 plates (Figure 4c) could be observed from 0W-PL-OMt plate by means of counting the numbers of surviving bacterial colonies on it (number of colonies is approximately 297). This result demonstrated that Mt modified with D1821 (0W-OMt) cannot facilitate ε-PL to intercalate into its interlayer space without ultrasonic treatment. However, the nutrient agar plate of 600W-PL-OMt was similar to PL-Mt30’s, and there was no *E. coli* survived on it, demonstrating the strongest antibacterial activity against *E. coli* compared with others PL-OMt. This may be explained by higher ε-PL content intercalated into the layer of 600W-PL-OMt with the assistance of ultrasonic treatment [23]. The same result was observed in the plates which incubated *B. subtilis*. This strong antibacterial activity of 600W-PL-OMt against *E. coli* and *B. subtilis* was consistent with previous studies [14,43]. However, the PL-OMt prepared at 1200W and 1800W almost did not exhibit antibacterial activity against *E. coli* and *B. subtilis*. The numbers of bacterial colony-forming units which survived on 1200W-PL-OMt and 1800W-PL-OMt plates were close to those of the control group and 1800W-OMt, and were all countless. This may be due to low amount of ε-PL intercalated into interlayers of 1200W-PL-OMt and 1800W-PL-OMt, and cannot work. These antibacterial tests results are in accordance with the ***Δ***d displayed in Table 2. The ***Δ***d can be representative of the amount of ε-PL intercalated into the layer of OMt after the secondary modification with ε-PL. Thus, 600 W is the most suitable ultrasonic power for preparation of antibacterial PL-OMt, and the 600W-PL-OMt are preferably compatible with organic and polymeric materials.

## 4. Conclusions

The physicochemical and antibacterial properties of PL-Mt and PL-OMt modified by D1821 and/or ε-PL were investigated. The XRD results of the PL-Mt revealed successful incorporation of the -PL, as evidenced by the increase in the Mt d_001_-value from 1.22 to 1.79 nm. The FTIR spectra and TGA suggested that 6 g of ε-PL and 20 g of Mt was the optimal proportion of ε-PL/Mt. The OMt d_001_-values increased with an increase in the ultrasonic power, and their mass losses were in line with their d_001_-values. The PL-OMt d_001_-values increased further relative to those of the OMt, and reached the maximum of 3.83 nm for the 600W-PL-OMt.

However, the mass losses of PL-OMt did not change obviously with an increase in the ultrasonic power except for 0W-PL-OMt, demonstrating that the Mt interlayer space was limited, and the total modifier amounts that could be accommodated were fixed. Therefore, when greater amounts of D1821 were intercalated, lower amounts of ε-PL were penetrated. The antibacterial tests demonstrated that PL-Mt30 exhibited good antibacterial activity. Although 0W-PL-OMt did not exhibit strong antibacterial activity, Mt modified with D1821 and ε-PL with the assistance of the ultrasonic treatment of 600 W exhibited good antibacterial activity as PL-Mt30. Because of the wide antimicrobial spectrum of -PL, it can be speculated that PL-Mt30 and 600W-PL-OMt might display good antibacterial activity, not only on Gram-positive or Gram-negative bacterial, but also on yeast and fungi. These two types of antibacterial montmorillonites, where one is hydrophilic (PL-Mt30) and another is hydrophobic (600W-PL-OMt), can meet the different demands of application. Furthermore, these results of this study may provide a basis for the application of ε-PL as a Mt modifier. The combination of ε-PL and Mt may be further exploited as nanofillers for biopolymer reinforcement and antibacterial agents for food packaging.

## Figures and Tables

**Table 1:** Particle size and distribution of ε-PL with various concentrations.

ε-PL Concentration (g/100 mL Water)	Parameters	PDI
	Mean Diameter (nm)	
1%	1134.7 ± 53.4 ^c^	0.211 ± 0.167 ^a^
3%	1218.5 ± 98.7 ^c^	0.181 ± 0.102 ^a^
5%	1413.8 ± 68.8 ^b^	0.152 ± 0.107 ^a^
7%	1579.3 ± 21.9 ^a^	0.146 ± 0.050 ^a^

^a–c^ Different lowercase letters in the same column indicate significant differences (*p* < 0.05). The data represented in mean ± standard deviation (SD) (n = 3).

**Table 2:** d_001_-values of OMt and PL-OMt prepared at different ultrasonic powers.

Ultrasonic Power (W)	Interlayer Space (nm)		
	OMt	PL-OMt	*Δ*d
0	3.00	3.64	0.64
600	3.13	3.83	0.70
1200	3.13	3.66	0.53
1800	3.20	3.68	0.48

***Δ***d = d_001_-value of PL-OMt minus d_001_-value of OMt.
